# Counterparts

**Published:** 1887-07-09

**Authors:** 


### COUNTERPARTS.

By a Student of Eastern Philosophies.

(Concluded from p. 228, vol. ii.)

### Chapter V.—How it all ended.

No hope! no hope!

Dr. Owlett had put it less harshly, but this was the ultimate meaning of his words, and they rang in Archie's ears all through the dismal dinner-hour, as he sat silently opposite his mother, listening with apparent attention to the discussion of her autumn plans. At last the servants had left the room, and mother and son were alone.

"I really wish you would decide on something, Archibald," remarked Lady Frances. "Here is August already, and you have not made up your mind whether you will go abroad, or to Scotland, or to Branthamthorpe. I cannot think why you are staying in town so late."

Lady Frances knew perfectly well what was keeping her son in town.

"There is no hurry, mother," said Archie. "I expect I shall end in going down to Yorkshire; but don't let that interfere with any of your arrangements."

"If you are quite sure you will go to Yorkshire, I will see about inviting a few friends to stay with us—though it is rather late now. I should like Gertrude Manners to come; but I doubt if she will do so after your strange conduct."

"I have not done anything," said Archie, shifting uneasily in his chair. He did not like allusions to Miss Manners, with whom he had had a desultory kind of flirtation for the last four or five years.

"You have treated her in a most ungentlemanly manner," said his mother severely. "You have led her and other people to believe you cared for her, and by your marked attentions to her have kept other men off and spoilt her chances more than once. The only reparation you can make now is to ask her to marry you."

"Perhaps she would not have me."

"Perhaps not," replied Lady Frances, calmly.

Archie looked nettled, but did not answer.

"What am I to do?" asked his mother presently.

"About what?"

"About asking Gertrude to visit us. I do not think she will accept the invitation unless you come to an understanding with her first."

"She can stay away, then," said Archie, but he still looked vexed. Lady Frances rose, and came and stood by him with her hand on his shoulders.

"Archie," she said, "you know how near my heart this match between you and Gertrude lies; I planned it when you were babies together; will you not give your mother her own way in this one little thing?"

Lady Frances generally got her own way in everything, little or big.

Archie was touched. It was not often that his mother caressed him, and she had not called him "Archie" for years. He took her hand and drew it silently to his lips.

Again the words, "No hope!" sounded like a knell in his ears. What did it matter now whom he married? Edith was going to die. In any case it was Travers whom she loved; why should he lose both her and Gertrude? He was fond of Gertie; she was the nicest girl he knew next to Edith.

"I don't feel at all in the humour for proposing to anybody just now, mother," he said at last, with a heavy sigh.

"There is no occasion to do that quite yet; but if you really are serious in your attentions to Gertrude, I think I can manage everything satisfactorily, and you can do your love-making when it suits yourself."

Archie shuddered. It seemed horrible to be arranging marriage with another woman in this cold-blooded way while his own love lay dying.

"Do as you like," he almost groaned, as he rose and left the room hastily.

Lady Frances did as she liked; she was used to it.

Where was Paul's philosophy? Did he not rejoice that his twin soul could no longer be contaminated by her earthly union with a man unworthy of her? that she was already removed to that mysterious spirit-world for which he had so long striven to fit himself?

No; his grief was much like the grief of other men. He groaned aloud in his agony, and cursed the cruel fate which, in the shape of a foolish and ignorant old man, had brought her bright young life to an abrupt, untimely close. How willingly would he have given her of that full, healthy vitality which throbbed through all his pulses, and which, he remembered with sickening despair, would probably keep him alive through many long, weary years! But they would not let him come near her, and this was the end of it! She was dead!

Poor Paul had been driven to the verge of madness during the past fortnight. Having been, at Archie's instigation, denied admittance into the house where Edith lay ill, he had taken rooms opposite, and had spent his time in walking up and down the street, vainly seeking an entrance into the house by fair means or foul. The neighbours took him for a bailiff. He had written letters alternately imploring, persuading, and threatening to Captain Charteris, Mrs. Charteris, Dr. Owlett, and even to Archie Bannerman. Not the slightest notice had been taken of any of them. Could he have got hold of the servant, he would have tried the effect of bribery and corruption, but Bridget was too afraid of the evil eye to venture across the threshold of the door. Wild, impracticable thoughts of burglary and arson beset him; he longed to mesmerise the whole household, but none of them gave him the opportunity. O for that faith which could remove mountains! Paul would have been thankful for as much as would move a street-door.

And now it was all over! She was dead!

"Oh, my love! my love!" cried Paul, throwing his arms on the table in front of him, and burying his face in them, "would to God you had lived even to be the wife of another, or that I at least had died with you!"

"Paul! save me!"

What was that cry? whence did it come? What was that fleeting vision of a face which appeared for a moment but to vanish?

Paul sprang up with beating heart and dilated eyes, and looked all round. There was nothing. He listened intently. Silence answered him.

Moved by a sudden impulse, he hastened downstairs and across the street to the opposite house. To his surprise the door flew open as he touched it. Perhaps Bridget was keeping Miss Edith's wake, and had forgotten to secure it. Guided, as it seemed to him, by an unseen agency, Paul walked unerringly upstairs into Edith's room. It was empty, but for her. How fair she looked as she lay there in her coffin, with her lips slightly parted, her dark lashes sweeping her pale cheeks! Paul knelt down beside her and reverently kissed the cold, white hands, which were crossed on her bosom. He gazed long and earnestly at her face. A great calm had fallen on his perturbed and restless spirit. Was it to let him look on her earthly form for the last time that she had called to him from out of the invisible world and led his footsteps hither? or was the ghostly voice but a trick of his excited imagination? From what was he to save her? Surely nothing could harm her now!

Meditating thus, Paul half-unconsciously laid his hand on her head, and started suddenly; his delicate, nervous fingers had detected a slight warmth there.

"Edith!" he cried eagerly, "Edith!"

He still kept his hand on her head; his eyes blazed strangely; he was exerting the whole strength of his will to call her, as it were, from the dead. He felt as if all the vitality in his body were rushing from his finger-tips.

A faint sigh escaped from Edith's lips; slowly the long lashes raised themselves, and the dark grey eyes unclosed.

"Paul!"

He lifted her out of the coffin and held her tightly in his arms, unable to speak for joy. Edith could only clasp him round the neck and feel that life, *his* life, was surging through her veins, that he had brought her back from the brink of the grave, that they were united at last.

"Och! murdther! it's the corpse is aloive!"

The exclamation came from Bridget, who had heard a noise overhead, and whose curiosity had so far overcome her superstitious terrors as to induce her to investigate the cause of the disturbance. Her shrieks, when she recognised the man with the evil eye, soon brought the whole household to the spot.

"You see I am not dead after all, papa," said Edith, turning to her astonished and terrified parent as he stood thunderstruck in the doorway. "It was only catalepsy. I have been trying to tell you so for the last two days, but could neither speak nor move till touched by Paul's life-giving fingers."

Captain Charteris could only press her to his heart and declare he felt like Jairus, while Mrs. Charteris forgot her fear of infection, dropped the camphorated handkerchief from between her lips, and showered tears, kisses, and ejaculations upon her resuscitated daughter.

Suddenly Archie Bannerman stepped out from the darkness of the hall into the room, and his face, for the first time in his life, was deadly pale.

Edith looked frightened; she had forgotten all about Archie. Paul put his arm round her and drew her to his side.

"You have no claim on her now," he said; "she is mine."

"I know," said Archie hoarsely, with his eyes fixed on Edith's face, "I have forfeited my claim."

Then he turned and went heavily downstairs.

THE END.

